# It’s MORe exciting than mu: crosstalk between mu opioid receptors and glutamatergic transmission in the mesolimbic dopamine system

**DOI:** 10.3389/fphar.2014.00116

**Published:** 2014-05-27

**Authors:** Elena H. Chartoff, Hilary S. Connery

**Affiliations:** Department of Psychiatry, Harvard Medical School, McLean HospitalBelmont, MA, USA

**Keywords:** morphine, heroin, AMPA, NMDA, GluR1, opioid withdrawal syndrome, plasticity

## Abstract

Opioids selective for the G protein-coupled mu opioid receptor (MOR) produce potent analgesia and euphoria. Heroin, a synthetic opioid, is considered one of the most addictive substances, and the recent exponential rise in opioid addiction and overdose deaths has made treatment development a national public health priority. Existing medications (methadone, buprenorphine, and naltrexone), when combined with psychosocial therapies, have proven efficacy in reducing aspects of opioid addiction. Unfortunately, these medications have critical limitations including those associated with opioid agonist therapies (e.g., sustained physiological dependence and opioid withdrawal leading to high relapse rates upon discontinuation), non-adherence to daily dosing, and non-renewal of monthly injection with extended-release naltrexone. Furthermore, current medications fail to ameliorate key aspects of addiction such as powerful conditioned associations that trigger relapse (e.g., cues, stress, the drug itself). Thus, there is a need for developing novel treatments that target neural processes corrupted with chronic opioid use. This requires a basic understanding of molecular and cellular mechanisms underlying effects of opioids on synaptic transmission and plasticity within reward-related neural circuits. The focus of this review is to discuss how crosstalk between MOR-associated G protein signaling and glutamatergic neurotransmission leads to immediate and long-term effects on emotional states (e.g., euphoria, depression) and motivated behavior (e.g., drug-seeking, relapse). Our goal is to integrate findings on how opioids modulate synaptic release of glutamate and postsynaptic transmission via α-amino-3-hydroxy-5-methyl-4-isoxazolepropionic acid and *N*-methyl-D-aspartate receptors in the nucleus accumbens and ventral tegmental area with the clinical (neurobehavioral) progression of opioid dependence, as well as to identify gaps in knowledge that can be addressed in future studies.

## INTRODUCTION

Opioids comprise a class of endogenous, naturally occurring and synthetic compounds that bind to and activate one of three known opioid receptors: mu, delta, and kappa (MOR, DOR, KOR, respectively). All opioids possess analgesic properties, which humans have taken advantage of for thousands of years. They also have profound effects on physiology and mood that depend on the specific opioid receptor and site of action in the brain. Opiates, a subclass of opioids that are natural derivatives of the opium plant, *papaver somniferum*, include morphine and codeine, which are the two major metabolites of heroin. These compounds primarily activate MORs to produce euphoria that can motivate repeated self-administration, produce tolerance, dependence, and ultimately opioid addiction. One percent of all Americans meet criteria for having an opioid use disorder (OUD); heroin use has doubled since 2007, and 2% of all Americans age 12 and older report misuse of a prescription opioid analgesic within the past 30 days (NSDUH, 2013). In 2008, there were 15,000 accidental overdose deaths related to prescription opioid use alone (Center for Disease Control) and opioid analgesics are second only to marijuana as the first illicit drug reported taken by 1.9 million youth and older adult Americans (NSDUH, 2013). The partial MOR agonist buprenorphine combined with the diversion-preventing opioid receptor antagonist naloxone has been partially successful in engaging youth and adults with OUD into abstinence-focused treatment (Fudala et al., 2003; Mattick et al., 2008; Woody et al., 2008; Weiss et al., 2011). However, controlled data on longer-term outcomes is lacking and patients taking agonist therapies (e.g. the long-lasting, full MOR agonist methadone and buprenorphine) have high rates of relapse (>75%) upon medication withdrawal (Woody et al., 2008; Weiss et al., 2011).

In fact, the treatment course of OUD is primarily challenged by the experience of the opioid withdrawal syndrome (OWS), which is characterized by both a typical physical syndrome occurring acutely (24–48 h post-withdrawal) and also by an affective/cognitive syndrome of dysphoria, anxiety, irritability, and preoccupation with cravings to use opioids (Kreek and Koob, 1998). These affective withdrawal symptoms occur acutely, but they frequently have a protracted course in humans (Dole et al., 1966; Martin and Jasinski, 1969; see **Table 1**). Acute and protracted OWS is observed in controlled studies and in clinical practice to precipitate resumed opioid use; this is not only true for those first entering treatment and inexperienced in recovery practices but also true for those in longer-term recovery on agonist therapy who experience OWS during attempts to discontinue agonist therapy (Magura and Rosenblum, 2001; Woody et al., 2008; Weiss et al., 2011). Therefore, there is a great need to develop newer, medical therapies that are not pharmacologically based on opioids themselves to assist people with OUD in tolerating OWS without relapse to opioid use.

**Table 1:** Symptoms of unmedicated abstinence in heroin-dependent men*.

Days of abstinence		
Day 3	Day 10	Day 30
• Severe anxiety	• Moderate anxiety	• Mild anxiety
• Moderate depression	• Subclinical depression	• Mild depressive symptoms
• Highest craving	• Moderate craving	• Milder craving
• Nasal discharge	• Nasal discharge	• Nasal discharge
• Mydriasis			
• Abdominal pain			
• Diarrhea			
• Vomiting			

*Based on results of two studies reported by Li et al. (2009) and Shi et al. (2009); anxiety measured by the Hamilton Anxiety Scale (HAM-A), depression by the Beck Depression Inventory (BDI).

The affective/cognitive components of OWS may be the most important target for drug development, since non-opioid medications (e.g., adrenergic antagonists, anti-emetics, sedative-hypnotics) already exist and are widely applied to treat aspects of the physical syndrome. Research in rats demonstrates that naloxone-induced heightened acoustic startle, a pre-clinical proxy for anxiety sensitivity, persists up to 80 days following a single administration of morphine, whereas naloxone-induced conditioned place aversion is not seen after 20 days (Rothwell et al., 2012), suggesting that anxiety may be one of the most persistent protracted symptoms of the OWS. In addition, one clinical study in prescription opioid-dependent individuals suggests that when patients are blinded to buprenorphine taper schedules, their success rates in moving through opioid withdrawal to achieve sustained opioid abstinence may be improved (Sigmon et al., 2013). This could reflect a significant component of anticipatory anxiety about OWS under conditions where individuals are aware of forced reduction.

In order to most successfully treat affective/cognitive components of the OWS, it is imperative to understand how the normal brain processes rewarding and aversive stimuli to modulate behavior, and how opioids subsequently act to change behavior. Excitatory glutamatergic neurotransmission provides a basis for communication between neurons that enables behavior. Depending on the neural circuits activated, behavior can refer to anticipated stimuli, emotional response, learning (stimulus-response), or action – all of which become dysfunctional with addiction. The goal of this review is to present and synthesize the current state of knowledge on how activation of MORs modulates glutamatergic neurotransmission through α-amino-3-hydroxy-5-methyl-4-isoxazolepropionic acid (AMPA) and *N*-methyl-D-aspartate (NMDA) receptors. We will focus on MOR–glutamate interactions within the mesolimbic dopamine system, a key neural substrate for the affective consequences of acute and chronic opioids. The basic pharmacology, neuroanatomical localization, and physiology of MORs have been well studied in *in vitro* systems, animal models, and clinical research, and there are numerous comprehensive reviews describing these findings (Law et al., 2000; Williams et al., 2001, 2013; Shalev et al., 2002; Waldhoer et al., 2004; Bailey and Connor, 2005; Pasternak, 2012).

## MOR DISTRIBUTION AND ACTIONS

Mu opioid receptors are expressed throughout the brain. Several comprehensive studies have been published in which MOR binding sites are mapped (Mansour et al., 1988, 1994; Le Merrer et al., 2009). MORs are generally perisynaptic: they can be localized postsynaptically on dendrites and cell bodies where they regulate neuronal excitability and transduce receptor activation to downstream signal transduction pathways, and they can also be localized presynaptically on axon terminals where they inhibit neurotransmitter release via activation of K^+^ conductance and/or inhibition of Ca^2^^+^ conductance (Williams et al., 2001). The cellular and neuroanatomical distribution of MORs is critical for understanding the neural circuits involved in the initiation of opiate action and subsequent plasticity with chronic drug use.

In the context of opiate dependence and withdrawal, several key neuroanatomical substrates have been identified, in particular the reciprocal connections within the limbic subcircuit of corticostriatal circuitry: GABAergic neurons of the nucleus accumbens (NAc), dopaminergic neurons of the ventral tegmental area (VTA), and glutamatergic neurons of the prefrontal cortex (PFC). Importantly, these regions contribute to acute opiate reward, dependence, tolerance, somatic and affective signs of withdrawal, and relapse (Wise, 1989; Stinus et al., 1990; Harris and Aston-Jones, 1994; Bonci and Williams, 1997; LaLumiere and Kalivas, 2008; Chartoff et al., 2009; Shen and Kalivas, 2013). Rats will self-administer opiates directly into the VTA (Bozarth and Wise, 1983; Devine and Wise, 1994), which contains dopaminergic cell bodies, and into the ventral striatum NAc (Olds, 1982), which receives dopaminergic input from the VTA. Acute morphine increases dopamine release in the NAc (Di Chiara and Imperato, 1988b; Johnson and North, 1992) by inhibiting GABAergic neurons in the VTA and rostromedial tegmental nucleus (RMTg) that synapse on dopaminergic neurons (Tepper et al., 1995; Jalabert et al., 2011). Morphine dependence – characterized by physical and psychological withdrawal signs – is mediated by several brain regions, with the locus coeruleus and periaqueductal gray (PAG) region most sensitive to naloxone-precipitated somatic withdrawal symptoms (Koob et al., 1992). The mesolimbic system is also important for morphine dependence, with a key role in affective signs of withdrawal: microinjections of naloxone into the NAc causes conditioned place aversions (Koob et al., 1992), and administration of a dopamine D_2_-like, but not a D_1_-like, receptor agonist directly into the NAc attenuates somatic withdrawal signs (Harris and Aston-Jones, 1994). Also, dopamine release is decreased in the NAc during morphine withdrawal (Rossetti et al., 1992; Diana et al., 1995; Bonci and Williams, 1997), suggesting that the NAc may mediate certain aspects of morphine dependence. Other key brain regions important for opiate dependence include, but are not limited to, the amygdala, hippocampus, and bed nucleus of the stria terminalis (Mansour et al., 1995b; Gracy et al., 1997).

## MOR ACTIVATION AND INTRACELLULAR SIGNALING

The physiological effects of morphine are absent in mice lacking MORs (Matthes et al., 1996; Le Merrer et al., 2009), providing strong support for the idea that MORs are necessary for the clinically relevant effects of opiates. MORs belong to the G protein-coupled receptor (GPCR) superfamily of seven transmembrane receptors and the rhodopsin receptor subfamily and are linked to pertussis toxin-sensitive inhibitory heterotrimeric guanosine triphosphate-binding proteins (G_α__i_/G_α__o_). Overall, MORs, DORs, and KORs are approximately 60% identical to each other (Chen et al., 1993).

**FIGURE 1:**
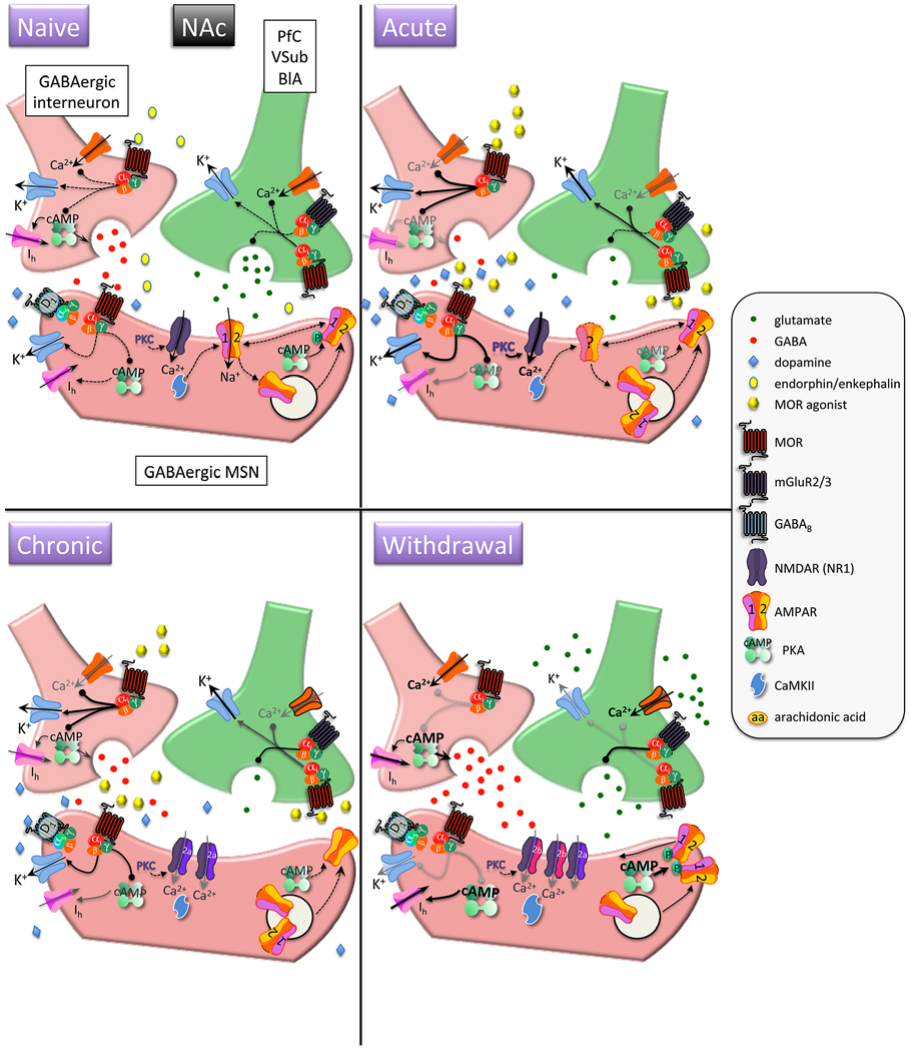
**Interactions between MOR and glutamatergic neurotransmission in the nucleus accumbens (NAc).** MORs are coupled to inhibitory G_α__i_ proteins and are found on glutamatergic and GABAergic terminals and postsynaptically on (primarily) D1 receptor-expressing MSNs. *Acute opioids*: Acute MOR activation in a naïve animal suppresses GABA and glutamate release via inhibition of Ca^2^^+^ and activation of K^+^ conductances, as well as inhibition of cAMP-mediated activation of non-selective cation pacemaker currents (*I*_h_). Postsynaptic NMDAR currents are augmented via MOR-induced PKC activation. There is no known data on the acute, immediate effects of opioids on AMPAR expression/localization/function in naïve animals. *Chronic opioids*: Inhibitory effect of presynaptic mGluR2/3 receptors to inhibit glutamate release is increased during chronic opioid treatment. Surface expression of GluR1 subunits is decreased on MSNs, with no change in total AMPAR subunit expression. Levels and/or function of the NR2A NMDAR subunit are increased, which may contribute to a decreased affinity for the co-agonist glycine and a decreased sensitivity to PKC-mediated NMDAR activation. *Opioid withdrawal*: Extracellular glutamate levels are increased, but synaptic transmission may be reduced via enhanced mGluR2/3 autoreceptor function. GABA release is potentiated via augmented cAMP and PKA pathways. NR2B surface expression is increased, perhaps resulting in an increase in silent synapses devoid of AMPARs. Upregulated cAMP and PKA signaling leads to increased P-GluR1^Ser845^, which may prime AMPARs containing GluR1 at the plasma membrane to be shuttled to synapse upon CaMKII activation. PfC, prefrontal cortex; VSub, ventral subiculum; BlA, basolateral amygdala; MSN, medium spiny neuron.

**FIGURE 2:**
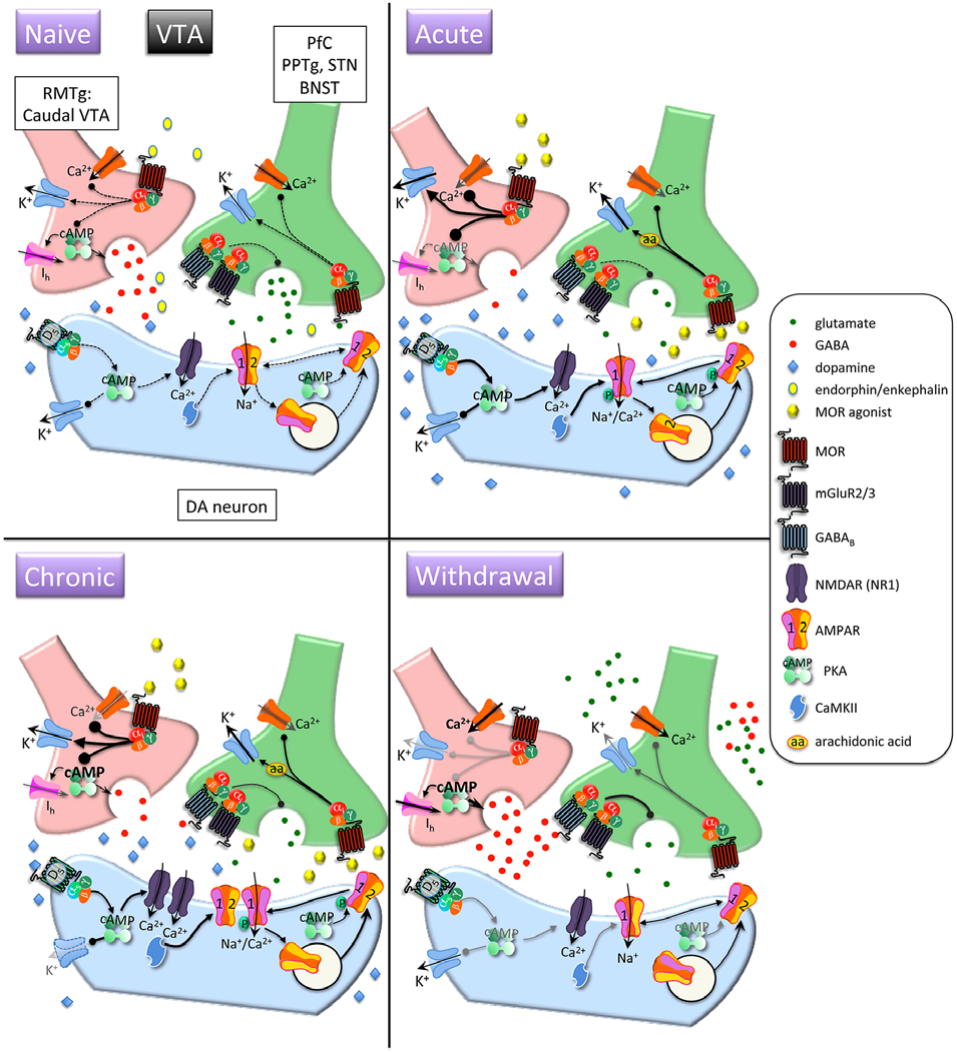
**Interactions between MOR and glutamatergic neurotransmission in the ventral tegmental area (VTA).** *Acute opioids*: Acute MOR activation in a naïve animal inhibits glutamatergic neurons via arachidonic acid-dependent potentiation of voltage-dependent K^+^ channels. GABA neurons are inhibited via G protein-mediated inhibition of Ca^2^^+^ and activation of K^+^ conductances, and GABA release is decreased via inhibition of cAMP-dependent facilitation of transmitter release. This leads to disinhibition of dopamine neurons and increased somato-dendritic dopamine release. Stimulatory dopamine D5 receptors on dopamine neurons are activated and, in conjunction with CaMKII, facilitate increased surface expression of GluR1 subunits. *Chronic opioids*: Dopamine firing rate remains elevated. Tolerance to inhibitory effects of MOR activation on GABAergic neurons develops through compensatory upregulation of cAMP systems, but dopamine neuron K^+^ channels are downregulated, enabling increased basal firing rate and burst activity of dopamine neurons. Total and surface GluR1 is increased and NR1 subunits are increased. *Opioid withdrawal*: Activity of GABA neurons is increased due to disinhibition of Ca^2^^+^ channels and reduced activation of K^+^ channels. GABA release is increased due to unmasking of upregulated cAMP systems. Extracellular glutamate levels are increased, but inhibitory presynaptic GABA_B_ and mGluR2/3 receptor function is enhanced, leading to decreased synaptic release of glutamate. RMTG, rostromedial tegmental nucleus; PPTg, pedunculopontine tegmental nucleus, BNST, bed nucleus of the stria terminalis; DA, dopamine.

Upon MOR activation, G protein α and βγ subunits interact with downstream effector systems to inhibit adenylyl cyclase and voltage-gated Ca^2^^+^ channels and to stimulate G protein-activated inwardly rectifying K^+^ channels (GIRKs) and phospholipase Cβ (Childers, 1991; Waldhoer et al., 2004; Williams et al., 2013; see **Figures 1** and **2**, Naïve condition, for depiction of MOR-dependent signaling). In the presence of chronic morphine, a compensatory increase in adenylyl cyclase activity occurs and cAMP levels return to normal (see **Figures 1** and **2**, GABAergic neurons in Naïve, Acute, and Chronic conditions). When morphine is discontinued or withdrawal is pharmacologically precipitated, cAMP levels dramatically increase (see **Figures 1** and **2**, GABAergic neurons in Withdrawal condition; Nestler and Aghajanian, 1997; Williams et al., 2001). This phenomenon of early inhibition and late positive regulation of adenylyl cyclase by morphine has been demonstrated in several morphine-receptive brain regions (Duman et al., 1988; Nestler and Tallman, 1988; Terwilliger et al., 1991; Van Vliet et al., 1991; Self et al., 1995; Shaw-Lutchman et al., 2002). Upregulation of the cAMP pathway observed during morphine withdrawal activates cAMP-dependent protein kinase A (PKA; Chartoff et al., 2003a, b, 2006). Interestingly, it has been reported that the increase in adenylyl cyclase activity may itself result in a decrease in transcript levels of particular cyclases in the striatum (Spijker et al., 2004).

**FIGURE 3:**
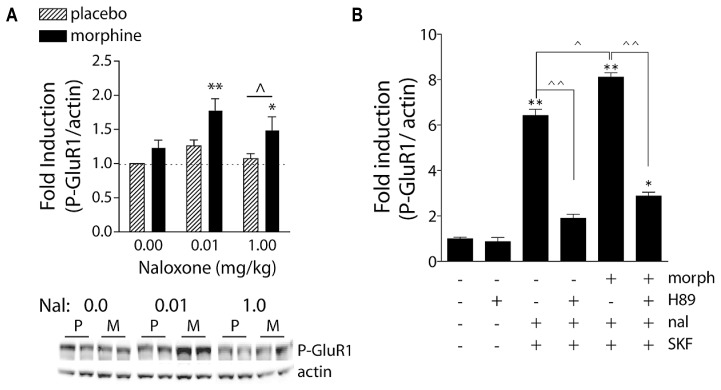
**Naloxone-precipitated morphine withdrawal increases GluR1 phosphorylation in a PKA-dependent manner.** **(A)** Rats were subcutaneously implanted with morphine (2 × 75 mg) or placebo pellets and returned to their home cages for 3 days in order for morphine dependence to develop. Naloxone (0.0, 0.01, or 1.0 mg/kg, SC) was injected and rats killed 30 min later. Brains were removed and frozen, 1-mm^3^ punches of the NAc were extracted, and P-GluR1^Ser845^ and β-actin (protein loading control) were quantified on immunoblots. Data are expressed as fold-induction of P-GluR1/actin levels relative to non-dependent (placebo) rats treated with vehicle. *p < 0.05, **p < 0.01 compared to non-dependent (placebo) rats treated with vehicle. ∧*p* < 0.05 comparing groups under bar. *N* = 5–9 rats/group. Modified from Chartoff et al. (2006). **(B**) PKA is required for super-induction of P-GluR1^Ser845^ during naloxone-precipitated morphine withdrawal. Primary striatal cultures were treated chronically with either vehicle or morphine (morph, 10 μM) for 6 days, followed by a 1.5-h treatment with vehicle [dimethylsulfoxide (DMSO)] or the PKA inhibitor H89 (20 μM), followed by a 30-min incubation with vehicle or naloxone (nal, 10 μM), and the dopamine D1 receptor agonist SKF 82958 (SKF, 50 μM) for 15 min. The ratio of P-GluR1^Ser845^/actin was determined for each sample and normalized to the control group ratio to yield a fold induction. Data are plotted as the mean fold induction ± SEM. *p < 0.05, **p < 0.01 compared with control. ∧*p* < 0.05, ∧∧*p* < 0.01 comparing groups designated by solid lines. *N* = 3 experiments with treatments in triplicate (see Chartoff et al., 2003a for details).

Protein kinase A phosphorylates and activates numerous substrates, including the transcription factor cAMP response element binding protein (CREB) and the AMPA receptor (AMPAR) subunit GluR1 (**Figure 1**, Withdrawal condition; **Figure 3**; Konradi et al., 1994; Chartoff et al., 2003b, 2006; Mangiavacchi and Wolf, 2004). Optimal PKA-mediated increases in CREB and GluR1 signaling requires NMDA receptor (NMDAR) activation (Konradi et al., 1996; Wolf, 2010), providing early evidence for crosstalk between MORs and glutamatergic transmission. It is through these actions that morphine and heroin may ultimately modulate fast excitatory transmission via AMPAR and NMDAR.

## GLUTAMATERGIC NEUROTRANSMISSION

The classic view of glutamate action comprises presynaptic release of glutamate, binding to postsynaptic ionotropic receptors, and clearance of glutamate by Na^+^-dependent glutamate transporters (Anggono and Huganir, 2012). Layered upon this are the more recently discovered influences of glial-derived glutamate release and uptake and extrasynaptic mGluRs on excitatory synaptic transmission and plasticity (Kalivas et al., 2009). Although this review focuses on MOR-mediated modulation of AMPAR and NMDAR-mediated glutamatergic transmission, it is essential to understand that glutamate homeostasis (regulation of synaptic and perisynaptic extracellular glutamate levels) requires ionotropic and metabotropic (mGluR) receptors as well as a delicate balance between glial and synaptic glutamate release and elimination. Comprehensive reviews of glutamate homeostasis in the context of drug addiction are available (Kalivas, 2009; Kalivas et al., 2009).

## AMPA RECEPTORS

AMPARs are a subgroup of ionotropic glutamate receptors found at most excitatory synapses, are activated at resting membrane potential, and are considered the primary postsynaptic mediators of glutamate transmission in the NAc (Cherubini et al., 1988). AMPARs comprise four subunits (GluR1–4) that assemble in various combinations to form tetramers (Seeburg, 1993; Hollmann and Heinemann, 1994; Dingledine et al., 1999). GluR1–4 share ~70% sequence homology and differ primarily due to post-transcriptional modifications, which confer unique properties to the subunits. For example, the GluR2 transcript undergoes RNA editing such that a glutamine residue in the channel-forming segment of the receptor is converted to an arginine (Sommer et al., 1991). This renders GluR2-containing AMPARs impermeable to Ca^2^^+^ (Burnashev et al., 1992). Given that AMPARs exist primarily as GluR1–2 and GluR2–3 populations (Wenthold et al., 1996), most AMPARs gate Na^+^ but not Ca^2^^+^. However, synaptic activity – including *in vivo* experience – can shift the stoichiometry of synaptic AMPAR subunit composition toward GluR2-lacking receptors (Liu and Cull-Candy, 2000; Takahashi et al., 2003; Ju et al., 2004; Clem and Barth, 2006), and increasing GluR1 expression favors formation of GluR1-homomeric AMPARs that allow Ca^2^^+^ flux (Hollmann et al., 1991).

Trafficking of AMPARs into and out of synapses determines the level of excitatory synaptic strength and is a major mechanism of plasticity underlying learning (Malinow and Malenka, 2002). AMPARs can be endocytosed and exocytosed into perisynaptic regions, and they can also be shuttled laterally along the surface of the neuronal membrane between synaptic and extrasynaptic compartments (Heine et al., 2008). A host of AMPAR auxiliary subunits such as transmembrane AMPAR regulatory proteins (TARPs), Cornichon proteins, Neuropilin, and Tolloid-like proteins (Netos) are necessary for the dynamics of AMPAR subcellular localization (Straub and Tomita, 2012). Heteromers containing GluR2–3 subunits are constitutively recycled and maintain basal AMPAR transmission, whereas heteromers containing GluR1–2 subunits are delivered to synapses in a precisely regulated manner and are critical for experience-dependent plasticity (see Malinow and Malenka, 2002). In the absence of activity, synapses can be devoid of GluR1–2-containing AMPARs. PKA-mediated phosphorylation of GluR1 at Ser^845^ (P-GluR1^Ser845^) enhances channel conductance and open probability, and in combination with activity-dependent Ca^2^^+^ signaling (e.g. via NMDARs), phosphorylation can drive GluR1 into synapses, which could allow synaptic strengthening (Esteban et al., 2003). Importantly, P-GluR1^Ser845^ is necessary but not sufficient for trafficking of GluR1 subunits to synapses (e.g. **Figure 2**, Chronic condition). In the NAc, this type of plasticity might involve convergence of dopamine and glutamate inputs (Wolf et al., 2003): activation of postsynaptic D1 receptors induces P-GluR1^Ser845^ and activation of NMDARs could allow synaptic delivery. Conversely, activation of AMPARs can lead to compensatory dephosphorylation of GluR1 and subsequent removal from synaptic zones to intracellular vesicles (Beattie et al., 2000; Snyder et al., 2003).

Synaptic scaling is a homeostatic form of plasticity in which prolonged activity or lack of activity at AMPARs (~1–3 days) leads to compensatory decreases or increases, respectively, in synaptic AMPAR levels (Turrigiano, 2008). This phenomenon is thought to stabilize neuronal activity during periods of abnormal or pathological activity, and may be highly relevant to addiction and drug withdrawal.

## NMDA RECEPTORS

NMDARs are a subgroup of ionotropic glutamate receptors found throughout the brain that act – in concert with colocalized AMPARs – as synaptic coincidence detectors to facilitate learning and memory (Tang et al., 1999; Citri and Malenka, 2008). NMDARs exist as heterotetramers composed of two NR1 subunits and two subunits from the NR2 or NR3 family (Seeburg et al., 1995). NR1 subunits are expressed ubiquitously in the brain, whereas NR2 subunits are spatially localized (Dunah et al., 1999). The basal forebrain (includes the NAc) is enriched for NR2A and B, with a predominance of NR2B in NAc medium spiny neurons (MSNs; Chen and Reiner, 1996; Kuppenbender et al., 2000). NMDARs are unique in that they require both ligand (glutamate) binding and membrane depolarization (to release extracellular Mg^2^^+^ block) in order to be activated. Once activated, NMDARs conduct both Na^+^ and Ca^2^^+^, which results in excitatory postsynaptic currents (EPSCs) with greater magnitude and longer half-life than those from AMPARs that pass only Na^+^. Perhaps most importantly, NMDAR activation engages Ca^2^^+^-mediated signal transduction pathways that can have long-lasting effects on gene expression, post-translational modifications of proteins (e.g., phosphorylation), and voltage-gated ion channels (Hyman et al., 2006). In fact, Ca^2^^+^ influx is required for NMDAR-mediated long-term potentiation (LTP). The NR1 subunit is essential for channel function whereas NR2 subunits control channel gating and Mg^2^^+^ dependency (Monyer et al., 1992).

Anatomical studies have shown that MORs and NMDARs colocalize on single neurons in many brain regions, including within the dorsal striatum and NAc shell (Trujillo, 2002; Glass et al., 2009). More recently, immunoprecipitation analysis has revealed that MORs can directly interact with NMDA NR1 subunits (Rodriguez-Munoz et al., 2012). This pattern was observed in the PAG, cerebral cortex, striatum, and dorsal spinal cord, suggesting functional interactions between MOR and NR1 are important for analgesic and affective responses to opioids.

The importance of NMDARs to opioid dependence and the OWS may lie in the well established role of NMDARs in forming associative memories via their ability to detect two coincident synaptic events at the cellular level (i.e., LTP; long-term depression, LTD). This type of learning is thought to be important for phenomena such as conditioned craving and conditioned withdrawal, which are common in abstinent opiate addicts and are major triggers of relapse. There is also evidence that NMDAR-mediated plasticity is necessary for extinction of drug-associated memories. Specifically, the NMDAR partial agonist, D-cycloserine (DCS) facilitates extinction of morphine withdrawal-associated place aversions in morphine-dependent rats (Myers and Carlezon, 2010a) and extinction of cocaine-induced conditioned place preferences (Botreau et al., 2006; Paolone et al., 2009).

Although NMDARs are classically thought of as a major substrate for Hebbian learning, they can also have unconditioned effects on reward and affective states. For example, rats will self-administer competitive and non-competitive NMDAR antagonists directly into the NAc (Carlezon and Wise, 1996b), and NMDAR antagonists potentiate brain stimulation reward (Carlezon and Wise, 1996a). These findings suggest that a reduction in the overall excitability of neurons in the NAc (via NMDAR blockade) and/or a reduction in intracellular Ca^2^^+^ signaling is sufficient for reward. It is likely that NMDAR-mediated increases in synaptic strength (learning) and changes in affective state are not mutually exclusive processes. One can envision a scenario during drug withdrawal in which the experience of an intense dysphoric state is stamped into memory through NMDAR activation in select brain regions. This idea will be discussed in more detail in the following sections.

## NUCLEUS ACCUMBENS

The NAc (ventral striatum) can be subdivided into multiple territories based on functional connectivity and neuronal phenotypes (Zahm and Brog, 1992). The NAc core is a central portion of the ventral striatum that surrounds the anterior commissure and is a functional continuation of the neighboring dorsal striatum. It has been shown to be particularly important for instrumental learning such as cue-induced reinstatement of drug seeking (McFarland and Kalivas, 2001). The shell comprises the most ventral and medial portions of the NAc, and has an important role in drug reward, motivated behavior, behavioral sensitization, and changes in affective state. In addition, subterritories such as the rostral pole, cone and intermediate zone of the NAc shell have been described (Zahm and Brog, 1992). A long-standing conception is that the NAc is a “motivation to movement interface” (Mogenson et al., 1980), and accumulating evidence has confirmed this idea by identifying the neural circuits that loop from limbic and cognitive cortical regions to motor output regions (Haber and Knutson, 2010). Thus, the NAc is a key site for transference of motivational and emotional signals to adaptive behavioral responses. Despite its long tenure as the “reward center” of the brain, increasing evidence supports the idea that the NAc is a bivalent structure that processes positive and negative emotional stimuli into either approach or avoidance behavior (Becerra et al., 2001; Reynolds and Berridge, 2002; Jensen et al., 2003; Roitman et al., 2005; Carlezon and Thomas, 2009). This has important ramifications for understanding addiction, since drugs of abuse provide hyperbolic positive (drug “high”) and negative (drug withdrawal, “crash”) emotional signals to the NAc.

### AFFERENTS

Consistent with the view that the NAc gates rewarding and aversive stimuli and directs subsequent goal-directed behavior, NAc afferents come from brain regions known to be important for processing both positive and negative emotional stimuli, such as the basolateral amygdala (Kelley et al., 1982) and for goal-directed behavior, including the orbitofrontal cortex, insula, cingulate cortex (Berendse et al., 1992), and midline and intra-laminar thalamic nuclei (Berendse and Groenewegen, 1990). In addition, the NAc receives rich innervation from the ventral subiculum of the hippocampus (Kelley and Domesick, 1982; Groenewegen et al., 1987), which likely provides spatial and contextual information about the stimuli (for review of NAc afferents, see Brog et al., 1993; Sesack and Grace, 2010). The vast majority of NAc afferents are glutamatergic and provide the excitatory drive necessary to evoke behavior. The NAc also receives some inhibitory, GABAergic inputs from the ventral pallidum and the VTA, as well as local inhibitory connections from within the striatum (Brog et al., 1993; Sesack and Grace, 2010). Layered on top of fast neurotransmission controlled by glutamate and GABA, the output of the NAc is modulated by robust networks of neuropeptides, both intrinsic and extrinsic to the NAc. These include, but are not limited to, orexin, dynorphin, enkephalin, substance P, and neurotensin (Hokfelt et al., 2000). Finally, dopamine afferents from the VTA provide an essential component of reward processing in the NAc. Dopamine modulates the general excitability of NAc neurons, thus increasing or decreasing behavioral output based on the level of emotional salience coded by the dopamine input (Koob, 1992; Ikemoto and Panksepp, 1999; Wise, 2004).

### INTRINSIC SIGNALING

Within the NAc, GABA-containing medium spiny output neurons comprise the majority (~90–95%) of neurons (Wilson and Groves, 1980; Gerfen, 1992), with the remaining cells being either GABAergic or cholinergic interneurons (Kawaguchi et al., 1995). The function of MSNs depends on their particular inputs and outputs, but also on the phenotype of the MSN itself. Only recently have researchers had the tools to begin to dissect the complex microcircuitry of the NAc. As with the dorsal striatum, NAc MSNs can be broadly divided into dopamine D_1_-like (includes D_1_ and D_5_ receptors) or dopamine D_2_-like (includes D_2_, D_3_, and D_4_) receptor expressing circuits (Gerfen et al., 1990; Lobo, 2009). MSNs express different constellations of neuropeptides, with dynorphin often co-expressing with dopamine D_1_ receptors and enkephalin with dopamine D_2_ receptors (Gerfen et al., 1990; Lobo, 2009). In the NAc, MORs are expressed primarily by dynorphin- and D_1_ receptor-expressing cells (Georges et al., 1999).

Glutamate neurotransmission is kept under tight control: too much or too little can have devastating effects (Kalivas, 2009), whereas stimulus-dependent changes in glutamatergic transmission are necessary for learning (Kauer and Malenka, 2007). Structurally, synaptic input to MSNs is arranged such that glutamatergic afferents synapse on dendritic spines and modulatory inputs such as dopamine make connections extra-synaptically on dendritic shafts. This triad of spine, glutamate synapse and dopamine synapse allows dopamine to modulate the general excitability of NAc neurons (Surmeier et al., 2007; Sesack and Grace, 2010). Glutamatergic activation of NAc MSNs is mediated primarily by AMPARs (Hu and White, 1996). Approximately 90% of AMPARs in the NAc are made of GluR1 and GluR2 or GluR3 containing tetramers with only about 6% being GluR1–3 complexes (Wolf, 2010; Reimers et al., 2011). There is some evidence for a very small percentage of AMPARs in the NAc to exist as GluR1 homomers. Functionally, this implies that the vast majority of NAc AMPARs conduct Na^+^ but not Ca^2^^+^, given that GluR2 renders AMPARs impermeable to Ca^2^^+^. NMDARs play a critical role in tagging connections that receive convergent glutamate and dopamine inputs. For example, cortical excitation of selected MSNs in the presence of dopamine would lead to an increase in synaptic strength in a two-step process: activation of postsynaptic D_1_ receptors induces PKA-dependent P-GluR1^Ser845^ and activation of NMDARs facilitates synaptic delivery of GluR1 (Wolf, 2010).

### EFFERENTS

The functional consequences of glutamate transmission in the NAc are being elucidated: in general, NAc neurons are activated in response to aversive stimuli and inhibited in response to rewarding stimuli (Peoples and West, 1996; Carelli, 2002; Roitman et al., 2005). GABAergic MSNs from the NAc project to the ventral pallidum, substantia nigra (SN), VTA, hypothalamus, and brainstem (Haber et al., 1990). There is topographical organization such that a medial (i.e., shell) to lateral (i.e., core/dorsal striatum) series of projection loops allows emotion-based information from limbic-associated structures to transfer to motor-related areas of the basal ganglia (Haber et al., 2000). Within these spiraling loops, some NAc outputs – particularly in the core – are functionally analogous to the direct and indirect pathways described for the dorsal striatum (Sesack and Grace, 2010). Activation of G_α__s_-coupled D_1_-like receptors stimulates production of cAMP and tends to excite MSNs that project directly back to the VTA and the ventral pallidum (direct pathway), whereas activation of G_α__i_-coupled D_2_-like receptors inhibits cAMP production and tends to inhibit MSNs that selectively project to the ventral pallidum (indirect pathway; Lu et al., 1998; Surmeier et al., 2007). Thus, cortical activation of the direct pathway leads to disinhibition of motor circuits that enable reward acquisition whereas activation of the indirect pathway inhibits motor circuits that are maladaptive (Mink, 1996).

## VENTRAL TEGMENTAL AREA

The VTA has been extensively studied for its role in reward and addiction. Opioids are self-administered directly into the VTA (Bozarth and Wise, 1981; Devine and Wise, 1994), while blockade of VTA MORs suppresses heroin self-administration (Britt and Wise, 1983). Intra-VTA morphine injections produce conditioned place preferences (Bozarth, 1987), enhance the rewarding impact of intracranial self-stimulation (Broekkamp et al., 1976), and reinstate extinguished lever pressing for heroin (Stewart et al., 1984). Dopamine neurons make up about 60–65% of the cells in the VTA, with GABAergic (~25%) and glutamatergic (up to 15%) neurons making up the rest (Swanson, 1982; Nair-Roberts et al., 2008). Most classes of drugs of abuse increase dopamine release in efferent targets of the VTA, including the NAc (Di Chiara and Imperato, 1988a). Comprehensive reviews of the role of VTA dopamine in reward function and addiction have been published (Berridge and Robinson, 1998; Wise, 2004; Fields et al., 2007; Ikemoto, 2007; Wheeler and Carelli, 2009; Salamone and Correa, 2012), with the emerging view that not only does dopamine mediate the positive reinforcing effects of drugs but it is also instrumental in learning how particular behaviors lead to reward or aversion (Volman et al., 2013).

### AFFERENTS

The VTA is regulated by an integrated network of excitatory inputs arising from the PFC, the pedunculopontine region (PPTg), the laterodorsal tegmentum (LDTg), and the sub thalamic nucleus (Grace et al., 2007). These connections are organized in the sense that glutamatergic inputs from the medial PFC (mPFC) synapse on dopamine neurons that project back to the mPFC but not on those that project to the NAc (Carr and Sesack, 2000). The VTA and the more caudal “tail” of the VTA (RMTg) receives GABAergic input from the lateral habenula, NAc shell, and ventral pallidum (Zahm and Heimer, 1990; Jhou et al., 2009). Importantly, the RMTg provides tonic GABAergic inhibition of VTA dopamine neurons that keeps them in a pacemaker-type firing pattern in the absence of stimulation (Bourdy and Barrot, 2012). The transition from pacemaker-like firing of dopamine neurons to burst firing, which is thought to represent a phasic dopamine response associated with reward and reward-related cues, requires glutamate input from the PPTg–LDT complex (Floresco et al., 2003; Lodge and Grace, 2006; Grace et al., 2007).

### INTRINSIC SIGNALING

GABA neurons of the VTA and RMTg express dense MOR mRNA and immunoreactivity (Mansour et al., 1988, 1995a; Garzon and Pickel, 2001; Svingos et al., 2001; Jhou et al., 2009). Morphine indirectly excites dopamine neurons via inhibition of these GABA neurons that synapse on dopaminergic dendrites in the VTA (Johnson and North, 1992; Jalabert et al., 2011). This disinhibition of dopamine neurons requires NMDAR and AMPAR activation (Jalabert et al., 2011). Taken together, the effects of opioids on VTA function involve a close interaction between postsynaptic MORs and glutamate signaling.

### EFFERENTS

There is a topographical organization to the VTA, with dopamine and GABAergic efferents having a medial to lateral projection to output structures such as the NAc, PFC, cingulate cortex, and basolateral amygdala (Ikemoto, 2007). In addition, there is a rostral to caudal organization in which the ratio of dopamine to GABA decreases caudally (Bourdy and Barrot, 2012). In broad terms, there are stronger drug reward associations in the caudal-medial versus anterior VTA (Ikemoto, 2007).

## ACUTE OPIOIDS

### CLINICAL DESCRIPTION

The National Survey on Drug Use and Health (NSDUH, 2013), the National Monitoring the Future survey study (Johnston et al., 2014), and the Columbia CASA report (The National Center on Addiction and Substance Abuse, Columbia University, 2011) provide consistently alarming trends of early age initiation of prescription opioid misuse (1.5% of children age 12–13 years old used in the prior month) and heroin use, with a national average age of opioid initiation between 22 and 23 years old. Teenagers report high availability of illicit opioids: 20 and 45% of high school seniors report it is easy to get heroin and prescription opioids, respectively (Johnston et al., 2014). Teens report using both to get high and to relieve tension, despite pervasive disapproval and perceived risk (The National Center on Addiction and Substance Abuse, Columbia University, 2011). Teens describe being high on opioids as, “the best feeling ever,” or, “I finally felt happy,” which is not different from the self-reported experiences of adult initiates.

### MOR–GLUTAMATE INTERACTIONS IN THE NAc (see Figure 1)

Acute administration of opioids activates MORs and increases extracellular dopamine in the NAc (**Figure 1**, yellow ovals; Di Chiara and Imperato, 1988a). However, dopamine is not necessary for the acute rewarding effects of opiates in non-dependent animals, as dopamine receptor blockade or 6-hydroxydopamine (6-OHDA)-mediated dopamine denervation of the NAc does not prevent heroin self-administration (Pettit et al., 1984; Gerrits and Van Ree, 1996). Even evidence demonstrating a requirement for the NAc in opiate reward and reinforcement is equivocal. For example, mice can learn to self-administer MOR agonists directly into the NAc (Goeders et al., 1984; David and Cazala, 2000), and yet intra-NAc morphine fails to produce conditioned place preferences in rats (Schildein et al., 1998). Lesions or inactivation of the NAc partially reduce opiate self-administration (Zito et al., 1985; Dworkin et al., 1988; Alderson et al., 2001), but it is difficult to interpret the meaning of these data on their own, since a decrease in the number of drug infusions at a single drug dose can mean either a decrease or an increase in the reinforcing efficacy of a drug (Mello and Negus, 1996). A study showing that NAc lesions reduced progressive ratio (PR) responding for morphine in rats (Suto et al., 2011) supports the idea that the NAc plays a role in the motivation to work for morphine. Yet direct infusions of MOR antagonists into the NAc actually increase heroin self-administration (Vaccarino et al., 1985), which the authors interpret as a decrease in the reinforcing efficacy of heroin driving increased drug-taking. Thus, the NAc can modulate opioid reward and drug-taking behavior, but its precise role is complicated by prior drug experience and method of administration.

Human imaging studies have generally shown that, in drug-experienced people, an immediate (i.e., during the “rush”) effect of opioid administration is an increase in regional cerebral blood flow in the anterior cingulate cortex, thalamus, and amygdala (Schlaepfer et al., 1998; Kosel et al., 2008). In contrast, after the initial “rush” has subsided and the longer lasting euphoric effects of acute opioids emerge, blood flow tends to be decreased (London et al., 1990; Denier et al., 2013). This is consistent with electrophysiological and neurochemical findings in rats, in which systemic morphine inhibits spontaneous firing of a majority of neurons in the mPFC (Giacchino and Henriksen, 1996). In many neurons, MOR-mediated inhibition of adenylate cyclase results in a decrease in cAMP-dependent activation of voltage-dependent *I*_h_ pacemaker currents (**Figure 1**, GABAergic neurons, Acute condition; Williams et al., 2001). A decrease in cAMP shifts the voltage dependence to more negative potentials, making it harder to depolarize the neuron. MOR activation suppresses basal and evoked increases in extracellular glutamate in the NAc and dorsal striatum (**Figure 1**, glutamate neurons, Acute and Chronic conditions; Desole et al., 1996; Enrico et al., 1998; Sepulveda et al., 2004). Although the functional consequences of changes in cerebral blood flow and cortical activation are not yet known, the findings suggest that opioid-induced reward is associated with decreased cortical activity and potentially decreased glutamatergic input to downstream NAc MSNs. Despite the evidence for opiate modulation of glutamate release in the NAc, there is relatively little data on the role AMPAR and NMDAR play in mediating the acute rewarding effects of opiates. This is surprising, given the previously discussed findings that acute opiates have profound effects on glutamatergic projections to the NAc and that the activation state of MSNs plays an important role in affect and emotional responses to stimuli (Roitman et al., 2005; Carlezon and Thomas, 2009). One prediction, based on synaptic scaling (Turrigiano, 2008), is that opiate-induced decreases in glutamatergic transmission to the NAc would result in increased surface expression of AMPARs. There are no known studies that address this prediction directly. Rather, there is evidence that expression of NMDAR and AMPAR subunits is decreased in the NAc core 3 days after acute morphine exposure (Jacobs et al., 2005). Similarly, there is one study that reports a decrease in surface levels of NAc GluR1 24 h after an acute morphine injection (Herrold et al., 2013). Unfortunately this time course does not actually reflect the acute rewarding effects of morphine but may rather reflect a state of acute withdrawal (Rothwell et al., 2012).

Using intracellular recordings from NAc slice preparations, it has been reported that acute MOR activation depresses NMDA and non-NMDA (presumably AMPA) excitatory postsynaptic potentials (EPSPs) in the NAc through a presynaptic mechanism involving reductions in spike-generated Ca^2^^+^ currents (Martin et al., 1997). A general effect of MOR activation that could account for this is inhibition of presynaptic voltage-gated Ca^2^^+^ channels (L-, N-, P/Q-, R-) through G_α__o_–βγ subunits (see **Figures 1** and **2**, presynaptic glutamatergic neuron, Acute condition; Law et al., 2000). Although this would predict a decrease in MSN activation, this study also demonstrated that postsynaptic NMDA currents were augmented via a protein kinase C (PKC)-dependent mechanism (**Figure 1**, postsynaptic GABA neuron, Acute condition). The ultimate consequences of these opposing MOR actions are still not fully understood. A more recent study in awake and behaving rats showed that a non-contingent injection of heroin produced a small decrease (not significant) in extracellular glutamate in the core in drug-naïve rats (LaLumiere and Kalivas, 2008). Taken together, the available data suggest that acute opiates decrease glutamate release in the NAc in non-dependent animals, which is consistent with the general finding that decreases in NAc MSN activation are associated with reward-like states (Carlezon and Thomas, 2009).

### MOR–GLUTAMATE INTERACTIONS IN THE VTA (see Figure 2)

An immediate effect of an acute opiate injection is inhibition of MOR-containing GABAergic neurons (**Figure 2**, GABAergic neuron, Acute condition) in the RMTg that make strong synaptic contacts on the soma and dendrites of dopamine neurons and a subsequent decrease in LTP of these GABAergic synapses (Johnson and North, 1992; Niehaus et al., 2010; Jalabert et al., 2011; Mazei-Robison et al., 2011). A second immediate effect is a presynaptic inhibition of glutamatergic afferents via MOR-mediated, arachidonic acid-dependent, activation of voltage-sensitive K^+^ channels (**Figure 2**, Glutamate neuron, Acute condition; Manzoni and Williams, 1999). This effect on glutamate release is confusing, because one would expect morphine to produce rapid activation of dopamine neurons through both inhibition of GABA inputs and excitation of glutamatergic inputs. In fact, it has been reported that an opiate-dependent increase in AMPAR activation in the VTA is required for disinhibition of dopamine neurons (Gysling and Wang, 1983; Di Chiara and Imperato, 1988a; Jalabert et al., 2011). Although not fully understood, the issue is likely related to timing.

An acute response common to most classes of drugs of abuse, including opiates, is an increase in AMPA transmission in dopamine neurons measured 24 h after acute administration of drug (Ungless et al., 2001; Saal et al., 2003; Brown et al., 2010). This is thought to be due to an increase in surface expression of AMPARs. Since acute morphine inhibits activity of glutamatergic afferent neurons (Giacchino and Henriksen, 1996; Manzoni and Williams, 1999), this observed increase in evoked AMPA transmission may not immediately translate to increased excitation of the VTA. Rather, morphine-induced decreases in glutamate release to the VTA may promote compensatory, postsynaptic increases in AMPA signaling that produce LTP at select synapses. Consistent with this, an increase in surface expression of GluR1 in the VTA (**Figure 2**, Dopamine neuron, Acute condition) has been reported 24 h (Brown et al., 2010), and as early as 1 h (Lane et al., 2008), after morphine injection. The mechanism through which morphine increases GluR1 synaptic insertion and LTP is not fully understood, but is thought to involve stimulation of dopamine D_5_ receptors (**Figure 2**, Dopamine neuron, Acute condition), which belong to the Gαs-coupled D1-like receptor family (Schilstrom et al., 2006; Brown et al., 2010). D_5_, unlike D_1_, receptors are expressed on dopamine neurons of the VTA (Weiner et al., 1991; Khan et al., 2000). Thus, morphine-induced dopamine release can stimulate D_5_ receptors in the VTA, which would activate cAMP-dependent processes including PKA-dependent phosphorylation of GluR1. Phosphorylation of GluR1 facilitates synaptic insertion and increases synaptic current (Kessels and Malinow, 2009), providing a potential mechanism for feed-forward enhancement of morphine’s actions on dopamine neurons. Consistent with this, overexpressing GluR1 AMPAR subunits in the VTA sensitizes rats to the locomotor effects of acute morphine and potentiates morphine-induced conditioned place preferences (Carlezon et al., 1997). Although GluR1 trafficking is evident after only one injection of morphine, it is thought that the cumulative effects of repeated morphine treatment are necessary for the plasticity in dopamine neuron excitability that contribute to the development of sensitization (Carlezon and Nestler, 2002).

A critical role for VTA AMPA and NMDARs in acute opiate reward has been demonstrated in behavioral studies. Intra-VTA delivery of an NMDAR or an AMPAR antagonist increased heroin self-administration in the same manner that decreasing the available dose of heroin does (Xi and Stein, 2002). This is consistent with the idea that NMDA and AMPAR activation is necessary for the acute reinforcing effects of opioids. Intra-VTA blockade of either NMDAR or AMPAR decreases both the acquisition and expression of morphine conditioned place preferences (Harris et al., 2004). Place conditioning depends upon an associative memory of the pairing of an affective state (reward or aversion) with a context. Thus, the role of VTA glutamate transmission in opiate effects could be to promote associative learning and/or to promote a rewarding state that has salience as an unconditioned stimulus in the place conditioning paradigm.

## CHRONIC OPIOIDS

### CLINICAL DESCRIPTION

Most individuals who are recently opioid-dependent are not fully aware they are “hooked.” Getting high is still euphoric, and mild withdrawal symptoms are surprising and manageable. Cognitive appraisal is, “I can stop if I need to.” Seeking a more intensive high may lead the individual to change to a route of administration that produces a more rapid and potent effect (e.g. oral to intranasal, or intranasal to intravenous), to try a more potent formulation (switching analgesics or getting a “good batch” of heroin), or to mixing opioid use with other substances, particularly sedative-hypnotics.

Opioids can be administered chronically in a number of ways (steady dosing of extended release painkillers, repeated intermittent abuse, binge-type self-administration, or any combination of the preceding), which likely influences the resulting neural adaptations. As discussed above, an additional consideration in interpreting data from chronic drug studies is the time point at which molecular or behavioral measures are taken. Effects observed 24 h or more after the end of a chronic drug regimen may more accurately reflect drug withdrawal rather than chronic effects *per se*. Furthermore, accumulating evidence suggests that GPCRs (e.g., MOR) modulate synaptic activity on a timescale that extends well beyond that of initial receptor activation, resulting in a metaplasticity that can either lower or raise the threshold for induction of LTP-like processes (Tenorio et al., 2010).

### MOR–GLUTAMATE INTERACTIONS IN THE NAc

Chronic activation of MORs triggers counteradaptions in cAMP signaling such that adenylate cyclase function is enhanced (Nestler and Aghajanian, 1997; Williams et al., 2001). The presence of chronic opioids masks the effects of increased cAMP, but it alters how other GPCRs signal through adenylate cyclase. As one example, chronic morphine increases the inhibitory efficacy of presynaptic mGluR2/3 receptors on glutamate release in the NAc (**Figure 1**, Glutamatergic neuron, Chronic condition; Martin et al., 1999). This may evolve from an increased functional connectivity between mGluR2/3 receptors and upregulated cAMP signaling.

Numerous studies have examined effects of repeated psychostimulants (contingent and non-contingent administration protocols) on AMPAR-mediated synaptic transmission in the NAc (Wolf et al., 2004; Luscher, 2013; Pierce and Wolf, 2013). Unfortunately, relatively little is known about effects of chronic opioids. These types of studies are important, because available research has shown that cellular and structural consequences of opioids often differ from those of psychostimulants (Badiani et al., 2011). Chronic, steady state levels of morphine achieved with subcutaneous morphine pellet implants do not change total protein levels of AMPAR subunits (Chartoff et al., 2006), although this does not take into account changes in subcellular localization. To address this, Glass et al. (2008) used immunogold ultrastructural analysis to demonstrate that surface expression of GluR1 subunits is decreased after chronic morphine treatment (1 h after 14 days of non-contingent injections; **Figure 1**, postsynaptic GABA neuron, Chronic condition). This effect was localized to dopamine D1 receptor-expressing neurons in the NAc shell and in all MSN types in the core. In contrast, a different opiate regimen (1 day after 1 injection/day for 3 days) produced no change in subcellular distribution of either GluR1 or 2 in the NAc (Mickiewicz and Napier, 2011). The mechanisms by which chronic opioids modulate subcellular distribution of AMPAR subunits are not known. However, given that activation of MORs inhibits adenylate cyclase and cAMP production, it is possible that the resulting brake on PKA function leads to decreased P-GluR1^Ser845^ in the NAc (**Figure 1**, postsynaptic GABA neuron, Chronic condition), which would favor internalization processes (Song and Huganir, 2002; Mangiavacchi and Wolf, 2004). This is consistent with formation of LTD, which requires clathrin-dependent endocytosis of postsynaptic AMPARs (Brebner et al., 2005).

More is known about the effects of chronic morphine on NMDAR-mediated synaptic transmission in the NAc compared to AMPA transmission. As discussed above, acute morphine’s actions in the NAc include presynaptic inhibition of glutamate release as well as a postsynaptic potentiation of NMDAR EPSPs via activation of PKC (Martin et al., 1997). Postsynaptically, chronic morphine appears to have several effects on NMDARs, including a decrease in affinity for the co-agonist glycine and a decrease in the sensitivity of PKC-mediated NMDAR activation. Using dissociated primary cultures of NAc neurons, it was shown that these effects may be due, in part, to an increase in expression or function of the NR2A subunit (**Figure 1**, postsynaptic GABA neuron, Chronic condition; Martin et al., 2004). *In vivo* studies have reported increased protein levels of NR1 and NR2A in the NAc after chronic morphine (Inoue et al., 2003; Murray et al., 2007), although a separate study did not detect a change in NR2A (Bajo et al., 2006). An intriguing possibility for how MORs and NMDARs interact is described in the opioid pain literature. It has been reported that MORs and NR1 subunits physically associate in the periaqueductal gray (Rodriguez-Munoz et al., 2012). Although it is not known if this occurs in the NAc or VTA, it raises the possibility that MORs can have direct effects on glutamate signaling through G protein signaling and/or through direct interaction.

### MOR–GLUTAMATE INTERACTIONS IN THE VTA

Both basal firing rate and burst activity of VTA dopamine neurons are increased after acute, and during chronic, morphine treatment, resulting in elevated tonic levels of dopamine in the NAc (Leri et al., 2003; Georges et al., 2006; although see Mazei-Robison et al., 2011). However, an acute morphine challenge fails to further increase dopamine neuron activity (Georges et al., 2006), suggesting tolerance at the level of dopamine neuron activation. Recently it has been shown that chronic morphine increases intrinsic excitability of VTA dopamine neurons through downregulation of K^+^ channels (**Figure 2**, Dopamine neuron, Chronic condition) concomitantly with decreases in dopamine soma size (Mazei-Robison et al., 2011). Thus, dopamine neurons are more likely to fire, but because of their smaller size they release less dopamine. Taken together, these data raise the possibility that not only does chronic morphine maintain its inhibitory influence on GABAergic neurons in the VTA and RMTg, but it also increases the sensitivity of dopamine neurons to excitation (via *I*_h_). Chronic morphine increases total levels of GluR1 and NMDA NR1 subunits in the VTA (Fitzgerald et al., 1996), and ultrastructural analysis showed that surface GluR1 is increased in dopaminergic and non-dopaminergic neurons of the parabrachial and paranigral VTA (**Figure 2**, Dopamine neuron, Chronic condition; Lane et al., 2008). These findings could explain, at least in part, how postsynaptic glutamate transmission is augmented with chronic opioid treatment, and they also demonstrate that normal glutamatergic signaling is fundamentally altered. In the presence of morphine, arachidonic acid-dependent activation of voltage-dependent K^+^ conductances continues to reduce glutamate release from afferent terminals (Manzoni and Williams, 1999). Yet signaling through GluR1 and possibly NR1 subunits on postsynaptic cells is enhanced (LTP-like; Fitzgerald et al., 1996; Lane et al., 2008), with no evidence so far of changes in other AMPAR or NMDAR subunits.

The effects of these seemingly opposite phenomena on behavior are not well understood, and provide an important area of future investigation. Given that AMPARs lacking GluR2 subunits and NMDARs are able to pass Ca^2^^+^, the increase in VTA GluR1 and NR1 likely results in increased Ca^2^^+^-mediated signaling (**Figure 2**, Dopamine neuron, Chronic condition), which would be expected to selectively strengthen those synaptic connections that express elevated GluR1 and NR1. Consistent with this, intra-VTA infusion of NMDAR or AMPAR antagonists prior to morphine conditioning sessions or prior to tests for morphine conditioned place preferences blocked the development and expression, respectively, of place preferences (Popik and Kolasiewicz, 1999; Harris et al., 2004). These effects appear to be limited to the rostral VTA (enriched for dopamine neurons), as AMPAR blockade of the caudal VTA (enriched for GABA neurons) had no effect on morphine conditioned place preferences (Shabat-Simon et al., 2008). Also, mice with a global knockout of GluR1 show reduced naloxone-precipitated withdrawal signs after an escalating dose regimen of morphine (Vekovischeva et al., 2001). This suggests that GluR1 is required for full dependence to develop. Consistent with the idea that increased GluR1 expression in the VTA facilitates dopamine neuron activation, it was shown that transient over-expression of GluR1 using HSV (herpes simplex virus) vectors in the rostral VTA increased the rewarding effects of a morphine challenge, whereas GluR overexpression in the caudal VTA had the opposite effect (Carlezon et al., 2000).

## OPIOID WITHDRAWAL

### CLINICAL DESCRIPTION

With severe OUD, episodes of opioid withdrawal are more frequent and more aversive, and getting high gives only brief pleasure, mostly attributed to cessation of withdrawal. Individuals now use “to feel normal” and “to be able to function.” Pre-occupation with obtaining a steady source of opioids is now prevalent, and episodes of anxiety, irritability, and dysphoria are more frequent. This is a stage of securing a steady opioid supply through friends, dealers, or a doctor. This is also the stage when an individual who never intended to use intravenously converts to injection use.

Forced abstinence is accompanied by severe withdrawal, anxiety, dysphoria, and intense, recurrent cravings to use opioids. Those who cannot access a source become quite desperate to obtain opioids, and will frequently self-injure in order to receive opioid analgesia in emergency health care settings. The fear of being cut off from opioid supply becomes ever-present and motivation to hoard opioid supplies becomes habitual. This may present as routinely exploring medicine cabinets while visiting friends and family, and finding reasons to visit someone during illness or post-surgical recovery, in hope of surreptitiously taking narcotic analgesics from them. This may also be a time of criminal behavior initiation (stealing, prostitution, running goods, etc.) to support an opioid habit.

Withdrawal from chronic opioids essentially unmasks all the neural adaptations the brain produced in its attempts to equilibrate in the presence of drug. Consequently, neural circuits regulating everything from gastrointestinal function to affective states are instantly unbalanced, and an OWS emerges. A primary cause of psychological withdrawal signs, which include anxiety, dysphoria, depression, and irritability, is thought to be the dramatic reduction in dopamine neuron firing and dopamine release in efferent targets (Diana et al., 1995). Neural circuits other than the mesocorticolimbic system also play critical roles in the OWS – both somatic and psychological. These include norepinephrine (NE; Weinshenker and Schroeder, 2007), corticotropin releasing factor (CRF; Contarino and Papaleo, 2005), orexin (Mahler et al., 2012), dynorphin (Yuferov et al., 2004; Schlosburg et al., 2013), and many more (for review, see Koob, 2009). It is likely that MOR-induced neuroplasticity in glutamate transmission underlies – at least in part – the effects of each of these systems on OWS.

### MOR–GLUTAMATE INTERACTIONS IN THE NAc

Spontaneous or naloxone-precipitated withdrawal from chronic opioids leads to a general increase in neuronal activity and transmitter release due to the removal of inhibitory MOR tone. For example, GABA release is increased in the NAc during withdrawal, particularly after activation of adenylate cyclase (**Figure 1**, presynaptic GABA neuron, Withdrawal condition; Chieng and Williams, 1998). Importantly, the ability of opioids to inhibit GABA release is also enhanced, suggesting that this may be one mechanism underlying the irresistible temptation to fight OWS with opioids themselves. Glutamate release has also been shown to increase, and numerous studies have shown that systemic or intracerebroventricular administration of NMDAR or AMPAR antagonists reduces morphine tolerance and/or withdrawal signs (Trujillo and Akil, 1991; Tokuyama et al., 1996; Gonzalez et al., 1997). Much less is known about the specific role of NAc glutamate transmission in the OWS. Extracellular glutamate levels are significantly increased in the NAc during morphine withdrawal (**Figure 1**, presynaptic Glutamatergic neuron, Withdrawal condition; Aghajanian et al., 1994; Desole et al., 1996; Sepulveda et al., 1998, 2004), although increased extracellular glutamate does not necessarily mean that excitatory synaptic transmission is increased (Kalivas, 2009). For example, it has been shown that presynaptic mGluR2/3 inhibitory autoreceptor function is increased during morphine withdrawal (**Figure 1**, presynaptic Glutamatergic neuron, Withdrawal condition) and mGluR2/3 receptor agonists attenuate behavioral signs of morphine withdrawal (Robbe et al., 2002b) and context-induced reinstatement of heroin seeking (Bossert et al., 2006). These findings support the idea that, although glutamate levels are increased, synaptic transmission may be decreased during withdrawal. Thus, it is not yet clear how the combination of chronic morphine-induced increases in AMPAR and NMDAR subunit expression, withdrawal-induced increases in extracellular glutamate, and increased autoinhibition of cortical afferents is synthesized into behavioral output.

Understanding the molecular mechanisms by which increased extracellular glutamate and AMPAR and NMDAR in the NAc contribute to OWS will be key to understanding and preventing relapse. As one example, the mechanism by which presynaptic mGluR2/3 receptor function is augmented is not known. Under normal conditions, these Gαi-coupled receptors inhibit evoked glutamate release by P/Q Ca^2^^+^ channel inhibition and PKA-dependent mechanisms (Robbe et al., 2002a). Chronic morphine and withdrawal has no effect on these processes in the NAc, raising the possibility of MOR-induced novel signaling mechanisms (Robbe et al., 2002b). In a second example, Shen et al. (2011) showed that 2 weeks of extinction following 2 weeks of daily heroin self-administration resulted in thinner dendritic spines in the NAc concomitant with an increase in surface expression of NR2B subunits (**Figure 1**, postsynaptic GABA neuron, Withdrawal condition). Consequently, overall synaptic strength was unchanged, but the AMPA/NMDA ratio (a proxy for synaptic plasticity) was decreased due to increased NMDA current with no change in AMPA current. What this means for the OWS was not investigated in this study, but Shen et al. (2011) found that the heroin withdrawal-induced increase in surface NR2B was necessary for heroin- and cue-induced reinstatement of heroin seeking. A heroin prime given to rats in which heroin seeking had been extinguished resulted in a rapid increase in spine density and synaptic strength. This group concluded that increased NR2B formed silent synapses in PFC to NAc core connections such that a reinstatement trigger enabled synapses to rapidly develop an LTP-like increase in field-potential strength necessary for resumption of heroin seeking. Based on prior studies of how silent synapses are “unsilenced” it is likely that Ca^2^^+^-induced Ca^2^^+^/calmodulin-dependent protein kinase II (CaMKII) facilitates shuttling of AMPARs from extrasynaptic sites on the plasma membrane to synaptic zones (Kerchner and Nicoll, 2008). In a different study, NR2A knockout mice treated chronically with morphine show reduced somatic withdrawal signs (Inoue et al., 2003). Restoration of NR2A expression selectively in the NAc allowed for the expression of somatic withdrawal signs. The NAc is not usually perceived as a substrate for somatic withdrawal, but this and other studies (Harris and Aston-Jones, 1994; Chartoff et al., 2009) indicate that it is a necessary – and perhaps even sufficient – component.

Morphine dependence and withdrawal may lower the threshold for LTP-like processes through a cAMP mechanism. Acute stimulation of Gαi-coupled MORs leads to a decrease in cAMP levels (Childers, 1991). In the presence of chronic morphine, however, molecular adaptations occur such that adenylate cyclase activity increases and cAMP levels return to approximately normal; when morphine is discontinued or the opioid receptor antagonist naloxone is administered, cAMP levels dramatically increase (for review, see Nestler and Aghajanian, 1997). Previously we have shown that naloxone-precipitated withdrawal increases levels of phosphorylated CREB (P-CREB) and P-GluR1^Ser845^ in the NAc of morphine-dependent rats (**Figure 3A**; Chartoff et al., 2006). Using primary cultures of dissociated striatal neurons, we demonstrated that administration of naloxone to cultures treated chronically with morphine enabled the dopamine D1 receptor agonist SKF 82958 to super-induce P-GluR1^Ser845^, an effect blocked by the selective PKA inhibitor, H89 (**Figure 3B**). Together, these data predict that surface expression (although not necessarily synaptic expression) of GluR1 subunits would increase during opioid withdrawal. This has not been directly tested, although it has been shown that targeted overexpression of GluR1 (but not GluR2) in the NAc produces anhedonia in the intracranial self-stimulation paradigm (Todtenkopf et al., 2006). One caveat may be that withdrawal-induced increases in extracellular glutamate trigger internalization/desensitization of AMPARs – reminiscent of synaptic scaling (Turrigiano, 2008). These predictions are not mutually exclusive, as glutamate-triggered desensitization would likely be a pan-NAc effect whereas PKA-mediated P-GluR1^Ser845^ and membrane insertion would likely occur only in MOR-expressing neurons.

As discussed in the beginning of this review, acute and protracted OWS has been shown clinically to precipitate relapse. Using an animal model of relapse, several studies have shown that glutamate release and AMPAR activation in the NAc core are necessary for reinstatement of heroin seeking after a period of withdrawal in which operant responding for heroin is extinguished (Bossert et al., 2006, 2011, 2012; LaLumiere and Kalivas, 2008). These studies raise an important issue – namely whether a heroin prime or a heroin-associated context (used as triggers for reinstatement) produces a negative affective state akin to OWS or a drug-like rewarding state that drives reinstatement. Increasing evidence supports the former: activation of NAc neurons (i.e., via glutamatergic transmission) is associated with aversive states (Carlezon and Thomas, 2009). The relevance of this hypothesis to heroin reinstatement studies remains to be tested.

### MOR–GLUTAMATE INTERACTIONS IN THE VTA

During morphine withdrawal GABA release is increased due to MOR-induced upregulation of cAMP signaling (**Figure 2**, GABAergic neuron, Withdrawal condition; Bonci and Williams, 1996, 1997), and glutamate release is decreased due to an increase in the potency of GABA_B_ receptor and mGluR-mediated presynaptic inhibition (**Figure 2**, Glutamate neuron, Withdrawal condition; Manzoni and Williams, 1999). Combined, these effects lead to a strong suppression of dopamine neuron activation (Diana et al., 1995). Interestingly, it has been found that chronic morphine’s almost ubiquitous upregulation of adenylate cyclase does not play a role in modulation of glutamate release in the VTA during withdrawal (Manzoni and Williams, 1999) leaving the mechanism for augmented inhibition of glutamate release unknown for now.

One confusing aspect of MOR–glutamate interactions in the VTA during opioid withdrawal is that the actual time course of withdrawal-induced effects on glutamatergic neurotransmission is not known. Putting together available data, the immediate effect of withdrawal is relief of MOR-mediated inhibition of glutamatergic and GABAergic afferents to dopamine neurons and an increase in glutamate and GABA release. Subsequently, glutamate and GABA engage the more slowly acting metabotropic mGluR2/3 and GABA_B_ receptors on glutamatergic terminals resulting in decreased glutamatergic synaptic transmission (**Figure 2**, Glutamate neuron, Withdrawal condition). The complexity of this scenario raises the possibility that plasticity within micro-regions containing MOR-expressing GABA and glutamate terminals that synapse onto dopamine neurons results in fine temporal and spatial control over synaptic communication. How this affects NMDAR and AMPAR function is not known, although one prediction is that AMPARs get promoted to synapses within micro-regions in which glutamate release is decreased and removed from synapses in which glutamate release is increased. This selective strengthening of synapses could provide a mechanism for associative learning that occurs with conditioned withdrawal (Myers and Carlezon, 2010b).

## RELAPSE

### CLINICAL DESCRIPTION

In humans, the risk for relapse decreases the longer a person remains abstinent. This is thought to be due, in part, to the fact that the most powerful motivation to relapse stems from the desire to alleviate the initial physiological withdrawal. Opioid agonist therapies are extremely successful in treating this phase of the OWS. Unfortunately, withdrawal from these medications also produces withdrawal signs that can trigger relapse. Furthermore, they do not treat other facets of abstinence, including cue reactivity.

Abstinent addicts are at high risk for relapse due to conditioned craving and withdrawal elicited by previously drug-paired cues (Wikler, 1973; O’Brien et al., 1986). In fact, heroin addicts report that the temptation or urge to use drug is elicited most powerfully by drug-paired cues (Heather et al., 1991). Incentive salience is the term that describes the unconscious and hypervigilant focus on rapid identification of any environmental cues that predict access to opioid using. During early recovery treatment, patients are taught to avoid high-risk “people, places, and things” to prevent cue-conditioned relapse. However, they are often baffled by their inability to reliably detect and avoid such triggers. This is because incentive salience is not a learned association within conscious awareness. A common clinical example would be that of an abstinent opioid addict being drawn to a person who is actively using while not recognizing that behavioral cues associated with that person’s drug use, and not his/her personality, are the source of interpersonal interest. On a positive note, cue reactivity wanes with time, and it appears as if the success of abstinence itself begins to provide a protective factor against relapse (see Epstein et al., 2006).

### MORPHINE–GLUTAMATE INTERACTIONS IN THE NAc

The majority of studies examining the role of NAc glutamatergic transmission in animal models of opioid relapse utilize the reinstatement model of drug seeking, in which the operant behavior producing contingent opioid administration is extinguished over time and then reinstated with non-contingent drug, cue, or stress presentation (Shalev et al., 2002). There is little data on how glutamatergic transmission regulates negative reinforcement mechanisms stemming from OWS. In a seminal study, LaLumiere and Kalivas (2008) demonstrated in rats that a non-contingent heroin prime or discrete cues previously paired with heroin infusions increased extracellular glutamate in the NAc core via increased synaptic transmission from PFC afferents. Intra-NAc core AMPAR blockade prevented reinstatement of heroin seeking. A separate study showed that microinjections of the mGluR2/3 receptor agonist LY379268 into the NAc shell, which inhibits evoked glutamate release from cortical afferents, reduced context-induced reinstatement of heroin seeking (Bossert et al., 2006). Interestingly, this group proposed that the reduction in heroin seeking was due to decreases in the motivational significance of the heroin context rather than to interference with memory retrieval. This is consistent with the idea that incentive salience underlies the power of a drug-paired cue to evoke drug-seeking behavior, and raises the possibility that heroin-associated cues increase synaptic glutamate release in the NAc thus producing an aversive state (Carlezon and Thomas, 2009; although see Stewart et al., 1984). Finally, chronic heroin self-administration increases NR2B subunits in the NAc (see Chronic Opioids). This is necessary for a heroin prime-induced increase in synaptic strength, dendritic spine enlargement, and reinstatement of heroin seeking after a period of extinction (Shen et al., 2011).

Although the VTA has been implicated in conditioned and unconditioned reinforcing effects of opioids (Stewart et al., 1984), and intra-VTA microinjections of the mGluR2/3 agonist LY379268 partially alleviate context-induced reinstatement of heroin seeking (Bossert et al., 2004), there is relatively little data on VTA glutamatergic transmission and relapse.

### IMPLICATIONS FOR MEDICATIONS DEVELOPMENT

Opioid dependence and withdrawal disrupts excitatory neurotransmission in reward-related brain circuits, which contributes to negative affective states associated with OWS and to corruption of motivated behavior away from natural rewards toward obtaining and taking drug. Efforts are underway to develop pharmacotherapies that target these aspects of addiction, but there has not been a major advancement in treatment options. Given what is known about the effects of MOR activation on glutamatergic transmission within the mesolimbic dopamine system, some ideas for targets emerge (see **Figures 1** and **2**).

• Extracellular glutamate levels are increased in NAc and VTA during opioid withdrawal.
• NMDAR levels/function is increased in both the NAc and VTA with chronic opioids.
• MOR-expressing neurons become hyperexcitable with opioid withdrawal.
• GluR1 AMPAR subunits decrease in NAc and increase in VTA with chronic opioids.

             Some compounds that act on these targets and have shown some promise in the treatment of addiction include:

• *Topiramate/Lamotrigine* – Used therapeutically as anticonvulsants and mood stabilizers. Mechanisms of action include inhibition of voltage-gated Na^+^ and Ca^2^^+^ channels and activation of GABA_A_ receptors (Rogawski and Loscher, 2004). May also block GluR5-containing AMPARs. Showed some promise as an adjunct during detoxification in a small study (Zullino et al., 2004).
• *Lacosamide* – Used therapeutically as an anticonvulsant. Mechanism of action is to enhance slow inactivation of voltage-gated Na^+^ channels (Beyreuther et al., 2007). Reduces the reward-related effects of cocaine at doses that do not impact motor capacity (Beguin et al., 2012).
• *Memantine* – Used to treat cognitive decline in Alzheimer’s patients. Primary mechanism of action is as a noncompetitive NMDAR antagonist. Reduced expression of naloxone-precipitated physical withdrawal signs in heroin-dependent patients (Bisaga et al., 2001).

None of these compounds have demonstrated remarkable effects, indicating that specifically targeting glutamate transmission will not be a panacea for opioid addiction. Notably none have been tested on protracted withdrawal signs such as anxiety and depression or on conditioned withdrawal or craving.

## CONCLUSION

Mu opioid receptor agonists such as morphine and heroin perturb the delicate balance of neurophysiological communication maintained by endogenous opioid peptides in the brain. The fact that a heroin “rush” or naloxone-precipitated withdrawal signs in opiate-dependent individuals can be felt within seconds of intravenous injection is evidence that the onset and offset of MOR activation can have rapid effects on cellular activity. The fact that some people develop a loss of control over opiate intake such that they engage in compulsive drug taking behaviors – despite severe negative consequences – is evidence that activation of MOR-coupled G proteins can have slower effects to alter neural circuits regulating motivated behavior. And the fact that drug-associated cues or contexts trigger relapse at some point in almost all opiate addicts trying to stay abstinent is evidence that activation of MOR-coupled G proteins facilitates long-lasting synaptic plasticity that maintains drug-related memories. As discussed in this review, this constellation of MOR effects stems in a large part from crosstalk between MOR-associated G protein signaling and glutamatergic neurotransmission.

## Conflict of Interest Statement

The authors declare that the research was conducted in the absence of any commercial or financial relationships that could be construed as a potential conflict of interest.
